# Common intestinal parasitic infections among patients living in Riyadh, Saudi Arabia: Prevalence and demographic associations (A cross-sectional retrospective study)

**DOI:** 10.1016/j.amsu.2022.103677

**Published:** 2022-04-28

**Authors:** Yousra Eldaw Abdelkareem, Anwar H. Abohashem, Ziad A. Memish, Abdulwahab Z. Binjomah, Fatima M. Takroni, Hind S. Al-amoudi, Ashwaq H. Masluf, Saad M. Alsurayea, Nader Alharbi, Ibrahim M. Aldealej

**Affiliations:** a Pathology and Clinical Laboratory Administration, King Saud Medical City, Riyadh, Saudi Arabia; b Microbiology Department, Riyadh Regional Laboratory & Blood Bank, Riyadh, Saudi Arabia; c Research & Innovation Center, King Saud Medical City, Riyadh, Saudi Arabia; d College of Medicine, Alfaisal University, Riyadh, Saudi Arabia; e Hubert Department School of Public Health, Emory University, Atlanta, USA; f King Saud Bin Abdulaziz University for Health Sciences, King Abdullah International Medical Research Center, Riyadh, Saudi Arabia

**Keywords:** Intestinal parasite, Blastocystis hominis, Prevalence, Saudi Arabia, Nationality

## Abstract

**Background:**

This study aimed to determine the prevalence and associated factors of intestinal parasitic infections (IPIs) among patients referred from different primary healthcare centers (PHC) in Riyadh, Kingdom of Saudi Arabia.

**Material & methods:**

A cross-sectional retrospective study conducted at Riyadh Regional Laboratory (RRL). All stool samples that are requested for intestinal parasite detection by physicians from PHCs across the Riyadh Region during year 2020 are referred to the RRL. The data recorded included age, sex, nationality, PHC location, and the stool analysis result with the type of parasite detected.

**Results:**

The data of 1148 patients were collected and statistically analyzed. IPIs were present in 296 (25.8%) patients, among whom 40 were infected with more than one parasite. The rate of infection with intestinal protozoa (95.4%) was higher than that with intestinal helminths (4.6%). Sixty (17.4%) infections were caused by pathogenic intestinal parasites, including pathogenic protozoa and helminths. The most common pathogenic protozoa were Entamoeba histolytica/dispar, which represented 9.3% of all IPIs and 72.7% of infections caused by pathogenic protozoa. Saudi nationals were the predominant population infected with pathogenic protozoa (44.0%). *Ascaris lumbricoides* was the most common helminth infection (56.3%) among patients. Nonpathogenic IPIs were detected at a higher rate (82.6%) than pathogenic IPIs (17.4%), with the predominant protozoa being Blastocystis hominis (61.0%). A higher rate of IPIs was observed in expatriates than in Saudi nationals (229 [33.6%] vs. 67 [14.3%], respectively) (P = 0.0000).

**Conclusions:**

Among the 12 different nationalities in our study cohort, the prevalence was the lowest in Saudi nationals (14.3%). The prevalence of B. hominis was high in all areas and nationalities, affecting all age groups among the patients referred for stool analysis. The implementation of preventive measures and awareness programs regarding sanitation and personal hygiene are needed.

## Highlights

• Among 1148 patients who had stool samples collected and tested, Intestinal Parasitic Infections (IPIs) were present in 296 (25.8%), among whom 40 were infected with more than one parasite.
• The rate of infection with intestinal protozoa (95.4%) was higher than that with intestinal helminths (4.6%).
• The most common pathogenic protozoa were Entamoeba histolytica/dispar, which represented 9.3% of all IPIs and 72.7% of infections caused by pathogenic protozoa.
• *Ascaris lumbricoides* was the most common helminth infection (56.3%) among patients.
• Nonpathogenic IPIs were detected at a higher rate (82.6%) than pathogenic IPIs.
• A higher rate of IPIs was observed in expatriates than in Saudi nationals.

## 1 Introduction

Intestinal parasitic infections (IPIs) caused by pathogenic helminths and protozoan parasites are endemic worldwide. Approximately 3.5 billion and over 450 million people are affected or ill with parasitic infections, respectively [1]. The majority of the morbidity burden from infections caused by intestinal protozoa and soil-transmitted helminths (STHs) is carried by tropical and subtropical countries. IPIs are one of the major public health problems for school children in developing countries, and they are a challenge for healthcare managers [1]. Intestinal parasites are mainly transmitted via the fecal–oral route by contaminated food or water or by direct contact with contaminated substances or surfaces [2]. According to the World Health Organization (WHO), more than 24% of people worldwide suffer from helminth and protozoal IPIs, most of whom reside in developing countries [3]. The most endemic regions are Sub-Saharan Africa, Southeast Asia, China, South India, and South America [4,5]. STHs that cause IPIs include *Ascaris lumbricoides* (roundworm), Trichuris trichiura (whipworm), Necator americanus, and Ancylostoma duodenale (hookworm), infecting more than 1.5 billion people, which is considered a relatively high proportion of the global population [6].

On a national level, determining the prevalence and distribution pattern of intestinal parasites is the first essential step to setting up an effective control program and improving the health status of the population. IPIs are the most common infections that significantly contribute to enteric diseases in both healthy and immunocompromised individuals [7].

The diagnosis of IPIs involves the microscopic detection of protozoan trophozoites and cysts and helminth eggs and larvae in stool samples. Because the density of parasites in stool samples is usually low, formal saline sedimentation method is used to increase the yield for diagnostic tests. Direct wet-mount microscopy is useful for observing motile trophozoites, but it is not recommended alone for the detection of other life cycle stages of the organism [8,9].

A study in Riyadh [10] reported an infection rate of 32.2% in a random sample of households. The infection rate was higher in non-Saudis (42.2%) than in Saudis (57.8%). Regarding the expatriate population, the infection rate was higher among males (47.6%), urban residents (48.3%), single persons (46.9%), tanker water users (39.5%), and septic tank users (78.6%). Furthermore, ages <12 years, non-Saudi nationalities, an educational level below secondary school, tanker water usage, and open sewage disposal were associated with high rates of IPIs.

A study on local public hospitals in Hail [11], Northwestern Saudi Arabia, reported the overall prevalence of IPIs to be 45.4%, of which 33.8% of patients were infected with one or more intestinal protozoa (3.8% were infected with helminths, and 7.7% had a mixed infection with both helminths and protozoa). The most common intestinal helminth detected was A. duodenale (3.8%), followed by *A. lumbricoides*, Taenia spp., and T. trichiura (1.5% for each species). The coccidian Cryptosporidium parvum (19.2%) was the most common intestinal protozoan, followed by Entamoeba histolytica (16.2%), Giardia lamblia (11.5%), *E. coli* (3.9%), and Blastocystis hominis (2.3%). The prevalence of IPIs in females was significantly higher than that in males.

A study of the prevalence of IPIs among expatriate foreign workers aged 20–60 years in Madinah, Kingdom of Saudi Arabia (KSA) [12], reported a prevalence of 44.2% (females, 47.5%; males, 52.5%). Some were infected with two or three different types of parasites. Another study conducted in Makkah, KSA [13] reported an IPI prevalence of 6.2%. The majority of patients were infected by E. histolytica (4.7%), followed by G. lamblia (1.3%), whereas 0.02% were infected with A. duodenale. Parasitic infections were more common in non-Saudi patients than in Saudi patients (7.1% vs. 5.8%, respectively). There was no significant difference between males and females in terms of parasitic infections, but the prevalence of parasitic infections was higher in patients aged <5 years (9.1%), followed by patients aged 5–14 years (7.5%). A study in Riyadh, KSA, reported that the prevalence of IPIs caused by pathogenic and/or nonpathogenic or both types of parasites was low, with an overall percentage of 10.6% [8].

The KSA has a large expatriate workforce originating from countries where IPIs are endemic [14]. Thus, delineating the prevalence and demographic factors associated with IPIs in the KSA is vital for determining appropriate public health interventions.

## 2 Methods

### 2.1 Study design and data collection

All stool samples that are requested for intestinal parasite detection by physicians from healthcare centers across the Riyadh Region are referred to the Parasitology Department at Riyadh Regional Laboratory (RRL). Data from RRL were retrospectively collected during year 2020, using Medisys, a web-based laboratory information system. The data recorded included age, sex, nationality, primary healthcare center location, and the stool analysis result with the type of parasite detected.

### 2.2 Stool sample analysis method

All stool samples of patients referred to RRL during 2020 were directly examined via wet-mount microscopy, concentrated using sodium acetate–acetic acid–formalin in the PARATEST®DK Diagnostics® kit, and stained using Wheatley trichrome staining.

### 2.3 Statistical analysis

The data were analyzed using SPSS statistical software v25.0 for Windows (IBM Corp., Armonk, NY, USA). Categorical variables (sex, nationality, and primary healthcare center area) were presented as frequency and percentage, and continuous variables (age) as mean ± standard deviation, range, and 95% confidence interval. Kolmogorov–Smirnov test was used to examine the normality of the values. The parametric test *t*-test was used to determine significant differences between two groups. Mann–Whitney *U* test, chi-squared test, and Fisher's exact test were used to examine the differences between two independent samples. A p-value of <0.05 was considered statistically significant for all tests.

Our work is fully compliant with the STROCSS 2021 criteria www.strocssguideline.com [15]. The study was registered with a Research Registry UIN: researchregistry7688 https://www.researchregistry.com/browse-the-registry#home/approved by the King Saud medical City IRB committee.

## 3 Results

During year 2020, the Parasitology Department at RRL received 1148 stool samples for intestinal parasite examination. Of them, 677 (59%) were from females and 471 (41%) were from males. The ages of the patients ranged from 1 to 87 years, with the majority aged 25–39 years (mean ± SD, 31 ± 14.6 years). (Table 1).

**Table 1:** Demographic information of 1148 patients.

Demographic Variables	Frequency positive patients (*n* = 296)	Frequency Negative patients (*n* = 852)	Total patients	prevalence (%) among all patients (1148)	Measure of association (χ^2^)	P-value	Odds Ratio	95% CI
Gender								
Male	109	362	471	23.1%	2.9127	0.0878	–	–
Female	187	490	677	27.6%*				
***Nationality***								
Saudi	67	400	467	14.3%	53.8139	0.0000*	2.34	
Expatriates	229	452	681	33.6%*				1.83–2.99
***Location***								
Central	178	495	673	26.4%	0.0079	0.9293	–	–
South	93	289	382	24.3%	0.2577	0.6117	–	–
West	25	68	93	26.9%*	Reference		1	–
***Age Groups***								
Mean ± SD (range)	30.79 ± 11.79 (1–86)	31.13 ± 15.5 (1–87)			t = 0.342	0.732		
<10	18	82	100	18%	0.000	1.000		
10-24	75	143	218	34.4%*	5.0856	0.0241*	1.9113	1.0288–3.5507
25-39	188	380	568	33.1%	4.8243	0.0281*	1.8388	1.0061–3.3608
40-59	55	157	212	25.9%	1.3828	0.2396		
>59	9	41	50	18%	Reference			
TOTAL	296	852	1148	25.8%				

*Statistically significant at 5% level.

The patients in our study cohort were referred from different healthcare centers distributed throughout three areas of the Riyadh Region: 673 (59%) from the Central area, which includes King Saud Medical City and other primary health centers, 382 (33%) from the Southern area, and 93 (8%) from the Western area. (Table 1).

The patient cohort included 467 (41%) Saudi nationals and 681 (59%) expatriates, with 12 different nationalities. The predominant nationality among the expatriates was Filipino (167, 24.5%), followed by Indian (128, 18.8%) (Table 1).

There were no significant differences between patients with and without IPIs and their sex and primary healthcare center location. However, significant differences in intestinal parasites and various demographic variables were observed in those aged 10–39 years and among expatriates. Overall, IPIs were present in 296 (25.8%) cases. Multiple infections were detected in 40 patients (13.5%) (up to four infections per patient), suggesting that the number of parasites detected was higher than the number of cases (296 patients infected with 345 parasites). The most common parasite causing IPIs was B. hominis (210, 61.0%), which overwhelmed all other types of organisms causing IPIs, with a wide spectrum of infection affecting all age groups across all study areas and among all nationalities. The second-most common IPI-causing parasite was Endolimax nana (36, 10.0%), followed by E. histolytica/dispar (32, 9.3%), *E. coli* (27, 8.0%), G. lamblia (12, 3.5%), Iodamoeba bütschlii (10, 3.0%), *Ascaris lumbricoides* (9, 2.6%), T. trichiura (4, 1.2%), Chilomastix mesnili (2, 1.0%), and Enterobius vermicularis, Schistosoma mansoni, and A. duodenale (1 case each, 0.3%) (Table 2).

**Table 2:** The infection rate of all parasites (pathogenic & nonpathogenic).

STOOL EXAMINATION RESULTS of 1148 PATIENTS			
Total number of examined patients	1148		
Number of Positive patients	296		
Total Number of parasite infections	345		
	#	Percentage	Prevalence
Patients with Multiple infection	40/296		13.5%
Protozoa	329		95.4%*
Helminthes	16		4.6%
Pathogenic parasites	60		17.4%
Non-pathogenic parasite	285		82.6%
Pathogenic protozoa	44/60	73.3%	
*Entamoeba histolytica*	32	72.7%	9.30%
*Giardia lamblia*	12	27.3%	3.50%
Nonpathogenic protozoa	285		82.6%*
*Blastocystis hominis*	210		61.00%
*Endolimax nana*	36		10.00%
*Entamoeba coli*	27		8.00%
*Iodamoeba butschlii*	10		3.00%
*Chilomastix mesnili*	2		1.00%
*Helminths (all pathogenic)*	16/60	26.7%	
*Ascaris lumbricodes*	9	56.3%	2.60%
*Trichuris trichuria*	4	25%	1.20%
*Enterobius vermicularis*	1	6.25%	0.30%
*Schistosoma mansoni*,	1	6.25%	0.30%
Hookworm	1	6.25%	0.30%

Of the 345 IPIs, 60 (17.4%) were caused by pathogenic intestinal parasites, comprising 44 (73.3%) protozoal infections (E. histolytica/dispar infection:(32, 53.3%) and G. lamblia infection: (12, 20%) and 16 (26.7%) helminth infections, including *Ascaris lumbricoides* (9, 2%) infection, T. trichiura (4, 6.6%) infection, and one case (1.7%) each of A. duodenale, E. vermicularis, and Schistosoma mansoni infections. Nonpathogenic protozoa comprised 285 of the 345 parasites (82.6%) and were predominantly B. hominis (210, 73.7%) (Table 2).

**Fig. 1:**
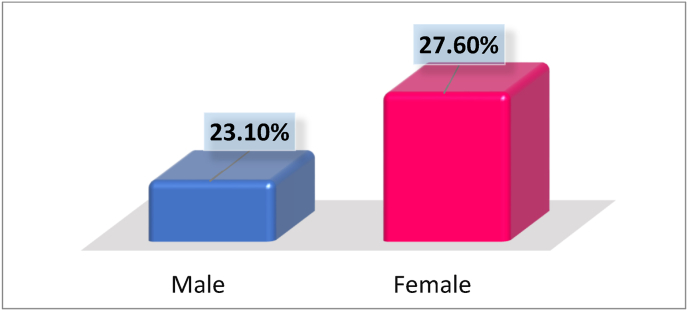
The prevalence of parasitic infections among gender.

The prevalence of IPIs was higher in females (27.6%) than in males (23.1%), and the age groups with the highest infection rates were 10–24 (34.4%) and 25–39 (33.1%) years (Table 2, Fig. 1).

**Fig. 2:**
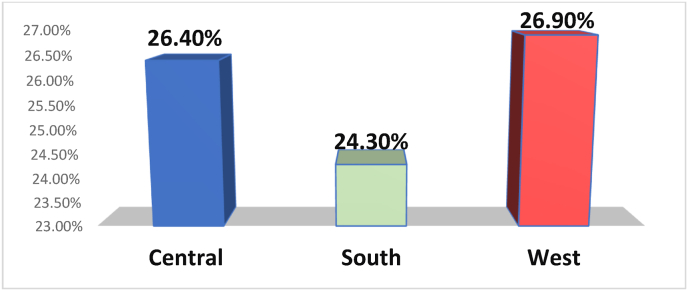
The prevalence of parasitic infections among different areas refers samples to Riyadh Regional Laboratory.

Our results revealed that the most infected areas were Western and Central Riyadh (26.9% and 26.4%, respectively), whereas Southern Riyadh had an infection rate of 24.3% (Table 1, Fig. 2).

A higher number of expatriates were infected (229, 33.6%) than Saudi nationals (67, 14.3%), and the prevalence of IPIs was the lowest in Saudi nationals among all nationalities. The highest prevalence of IPIs was in Pakistanis (58.8%), followed by Ethiopians (58.3%), Sudanese (48.5%), Kenyans (47.7%), Ugandans (40.8%), Sri Lankans (36.3%), Yemenis (34.8%), Indians (31.3%), Egyptians (30%), Filipinos (25.1%), and Bengalis (20%) (Table 3).

**Table 3:** The prevalence of parasitic infections among Saudi and different nationalities.

	Saudi	expatriates											
	Saudi	Bangladeshi	Egyptian	Ethiopian	Filipino	Indian	Kenyan	Pakistani	Srilankan	Sudanese	Uganda	Yemeni	TOTAL
Total + VE	67	14	15	7	42	40	41	10	5	16	31	8	296
total	467	70	50	12	167	128	86	17	19	33	76	23	1148
Prevalence%	14.3%	20%	30%	58.3%	25.1%	31.3%	47.7%	58.8%	36.3%	48.5%	40.8%	34.8%	%

Although the prevalence of IPIs was the lowest in Filipinos and Saudi nationals among all nationalities, Filipinos were infected with 11 of the 16 severely pathogenic intestinal helminths, and *A. lumbricoides* was the most prevalent among Filipinos (6 of 9 cases, 66.7%) compared with the other nationalities. Saudi nationals were infected with 14 types of pathogenic intestinal protozoa, and E. histolytica/dispar was the most prevalent among Saudis (14 of 32 cases, 43.8%) compared with the other nationalities (Table 4).

**Table 4:** Distribution of the 60 Pathogenic parasites among different nationalities.

	Bangladeshi	Egyptian	Ethiopian	Filipino	Indian	Kenyan	Pakistani	Saudi	Srilankan	Sudanese	Ugandan	yemeni	TOTAL
Alumbricoides	2			6 (66.7%)							1		9
Ehistolytica		2	1	2	3	7	1	14 (43.8%)		2			32
Evermicularis					1								1
GLamblia	1	1			6			2			1	1	12
Hookworm				1									1
Smansoni						1							1
Ttrchiura				4									4
Total	3	3	1	13	10	8	1	16		2	2	1	60

## 4 Discussion

This study explored the prevalence of IPIs and associated demographic factors among patients in Riyadh whose stool samples were submitted for analysis at RRL. A similar study conducted at RRL [8] using direct stool analysis and a formal ether concentration technique for only limited samples found an IPI prevalence rate of 7.6%, which is one-third of the prevalence rate reported in our study. All specimens in our study were tested as per the recommendations of the College of American Pathologists using a concentration technique and trichrome staining [16,17]. The use of a concentration technique for stool analysis was reported to increase the rate of parasite detection, with wet-mounts detecting only 57 IPIs, which increased to 69 IPIs when a concentration technique was used [12]. In our study, approximately one-fourth of the examined samples (25.73%) were infected with intestinal parasites. This finding corroborated the findings reported in other studies conducted in Riyadh and across different geographical areas in the KSA, which reported the highest prevalence in Madinah (44.2%) and the lowest prevalence in Makkah (6%) and Jeddah (5.3%) [10,12,13]. This is despite the fact that Riyadh the capital city of KSA has an advanced and well-established water distribution and waste management system.

The most commonly detected intestinal parasite in this study was B. hominis (210 cases, 61%), and its prevalence rate was higher compared with that reported in a study in Jeddah (32%) [18]. Our finding of a high prevalence of B. hominis requires further investigation because there is an ongoing controversy about the pathogenicity of B. hominis in certain individuals. Although some studies have demonstrated its nonpathogenicity, it has been attributed as the cause of watery diarrhea, fever, nausea, vomiting, anorexia, abdominal pain, irritable bowel syndrome, and colorectal cancer in other studies [[19], [20], [21], [22]].

It has also been hypothesized that a low level of personal hygiene and the presence of nonpathogenic parasites in the stool, indicating a reservoir of pathogenic intestinal parasites [23]. This was confirmed by another study, which found that E. histolytica and E. dispar infections were significantly associated with the presence of the nonpathogenic protozoa *E. coli* [24]. The most commonly detected pathogenic parasites in our study were E. histolytica/E.dispar (9.3%), affecting patients from 8 of the 12 nationalities. Furthermore, the highest infection rate of pathogenic protozoa was found in Saudi nationals (44%), followed by Kenyans (21.9%), and these findings were consistent with those of another study conducted in the Riyadh Region [25].

Our results showed that G. lamblia was the second-most prevalent pathogenic protozoa and that it mainly infected those of Indian nationality; this finding was consistent with the findings of a study in Hail, Northwestern Saudi Arabia, in which the prevalence of G. lamblia was reported as 28.5% among Indians, and G. lamblia and E. histolytica were the most prevalent protozoa across all nationalities [12].

We found that helminths tended to infect Asians. Filipinos were the predominant nationality infected with *A. lumbricoides* (66.7%), and the four cases of T. trichiura were detected in Filipinos. These results were consistent with the study conducted in Makkah [13] (Table 4).

The prevalence of IPIs was higher in females (27.6%) than in males (23.1%). Furthermore, IPI prevalence was highest among those aged 10–39 years (10–24 years, 34.4%; 25–39 years, 33.1%), which might be because the age group with the highest levels of soil-transmitted parasites were school children and also because that the majority of expats were aged 25–39 years. A study in Western Saudi Arabia among school children in Jeddah found that the prevalence of IPIs was 5.3%, with B. hominis being the most common parasite (32%) [17]. Our findings were similar to the findings of an Indian study, which reported that the highest rate of Ascaris spp. and A. duodenale infections occurred in those aged 26–30 years [26].

The prevalence of IPIs in expatriates (229, 33.6%) was significantly higher than that in Saudi nationals (67, 14.3%) because the majority of workers come to the KSA from tropical and subtropical areas, which are endemic areas for IPIs with a high prevalence of IPIs among the inhabitants [14]. Furthermore, the high prevalence of IPIs in developing countries compared with developed countries may be due to the contamination of food or water supplies and poor hygienic conditions [27].

## 5 Conclusions & recommendation

This study revealed a high prevalence rate of IPIs affecting the health of local and foreign populations as IPIs affect both school children and the most productive age groups. Our study revealed that pathogenic parasitic infections were mainly found among Saudi nationals. Thus, more community-based surveys and frequent follow-ups should be performed to confirm our data, in which case, identification of the source is needed as well as further education on the routes of transmission and improving hygiene practices. Preventive measures for school students and workers should also be mandatory to prevent the persistence of such infections.

The high prevalence of nonpathogenic parasites (82.6%) in Western and Central Riyadh in this study warrants further epidemiologic surveys and molecular studies to identify parasitic strains because the presence of nonpathogenic parasites in the stool indicates a reservoir of pathogenic intestinal parasites [23,24]. More expatiates were infected than Saudi nationals, and the analysis of one stool sample per patient may underestimate the actual prevalence rate [28]. Untreated or inadequate treatment of foreign workers with IPIs, particularly housemaids and food handlers, will continue to increase the prevalence of IPIs in the foreseeable future because these populations act as reservoirs of infection [29]. This fact and the results from other studies emphasize the necessity of applying sensitive protocols for pre-employment screening [28]. Three samples collected on three consecutive days, repeated treatment, and prophylactic treatments are recommended for all persons who have newly arrived from or have spent a holiday in an endemic area with positive IPI results.

## Ethical approval

KSMC IRB approval obtained.

## Sources of funding

None.

## Author contribution

All authors contributed equally.

## Registration of research studies

Name of the registry: research registry

Unique Identifying number or registration ID: 7688.

Hyperlink to your specific registration (must be publicly accessible and will be checked):

## Guarantor

Ziad A Memish.

## Consent

Exempted.

## Provenance and peer review

Not commissioned, externally peer reviewed.

## Declaration of competing interest

None.
